# Supplementary material for: Jaw shape and mechanical advantage are indicative of diet in Mesozoic mammals
Source: Commun Biol. 2021 Feb 23;4:242. doi: 10.1038/s42003-021-01757-3 (PMC7902851; doi:10.1038/s42003-021-01757-3)
Supplement: Supplementary file 2 — Supplementary Information [file 42003_2021_1757_MOESM2_ESM.pdf]

Supplementary Figures

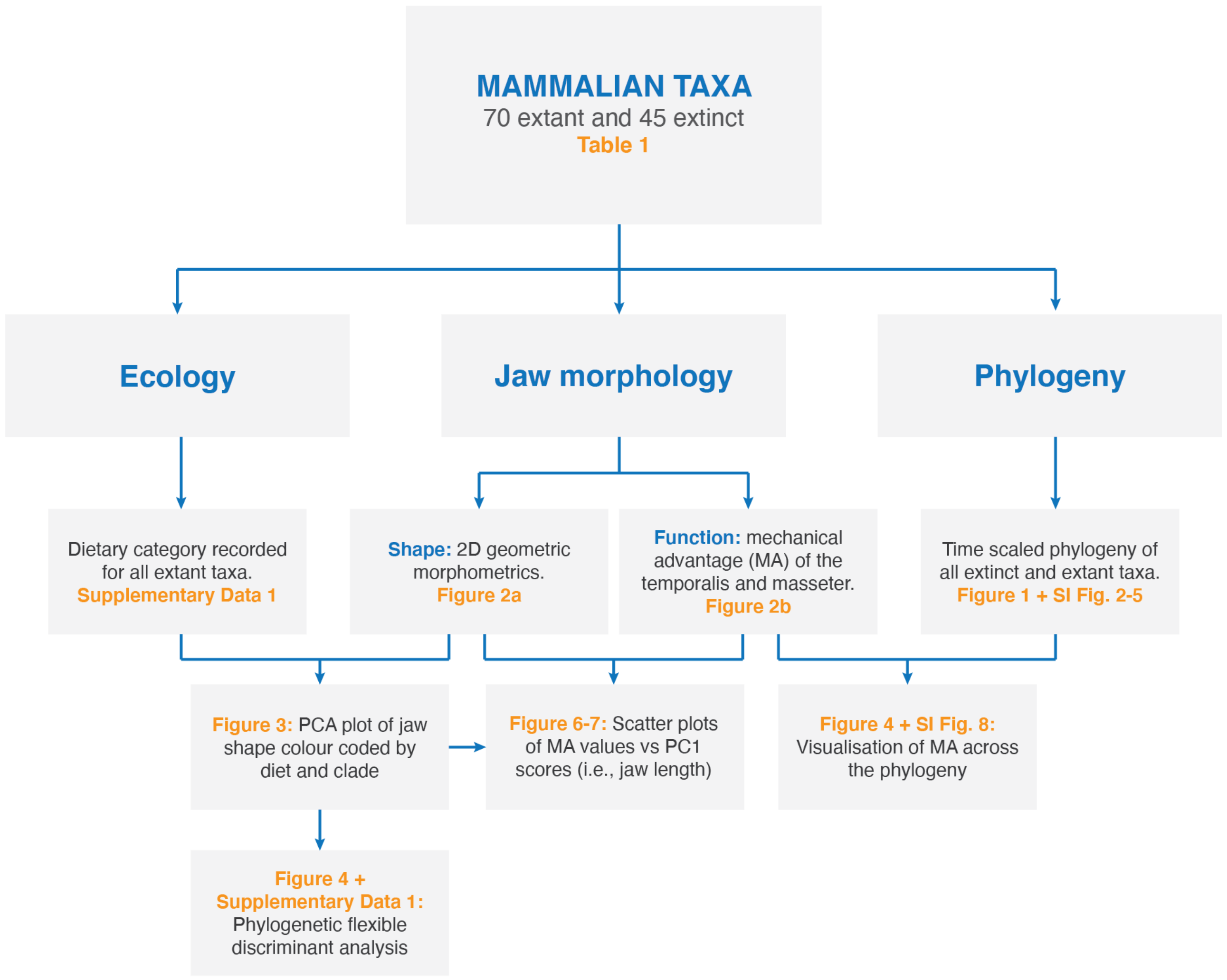

Figure 1: Summary of the methods and figures presented in this paper.

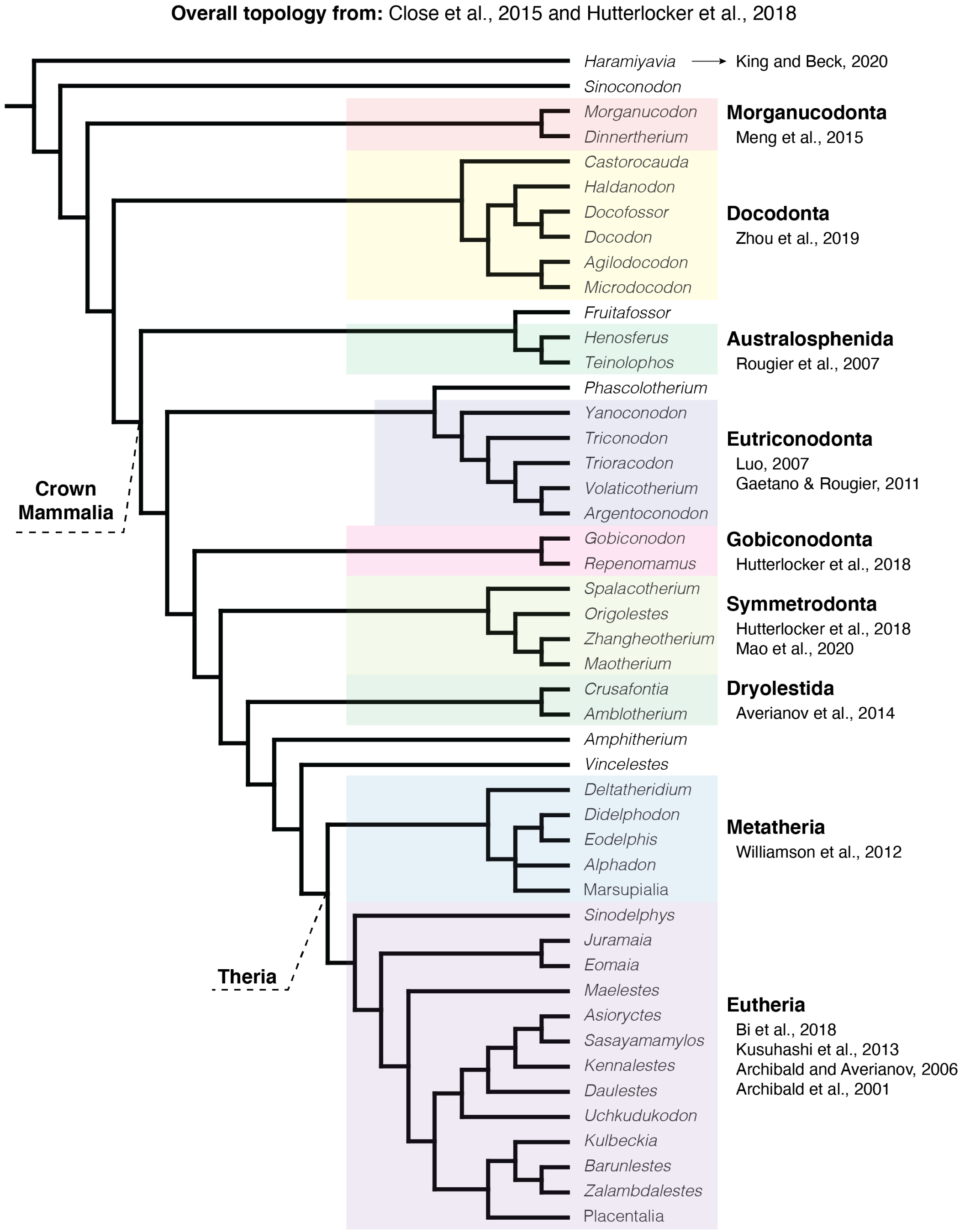

Figure 2: Genus-level phylogeny of the Mesozoic taxa used in this study. Phylogeny assembled from Close et al.[1] and Huttenlocker et al.[2] (overall topology), King and Beck[3] (*Haramiyavia*), Meng et al.[4] (Morganucodonta), Zhou et al.[5] (Docodonta), Rougier et al.[6] (Australosphenida), Luo[7], Gaetano and Rougier[8] (Eutriconodonta), Mao et al.[9] (Symmetrodonta) Avenianov et al.[10] (Dryolestidae), Williamson et al.[11] (Metatheria), Bi et al.[12], Kusuhasi et al.[13], Archibald and Averianov[14] and Archibald et al.[15] (Eutheria)

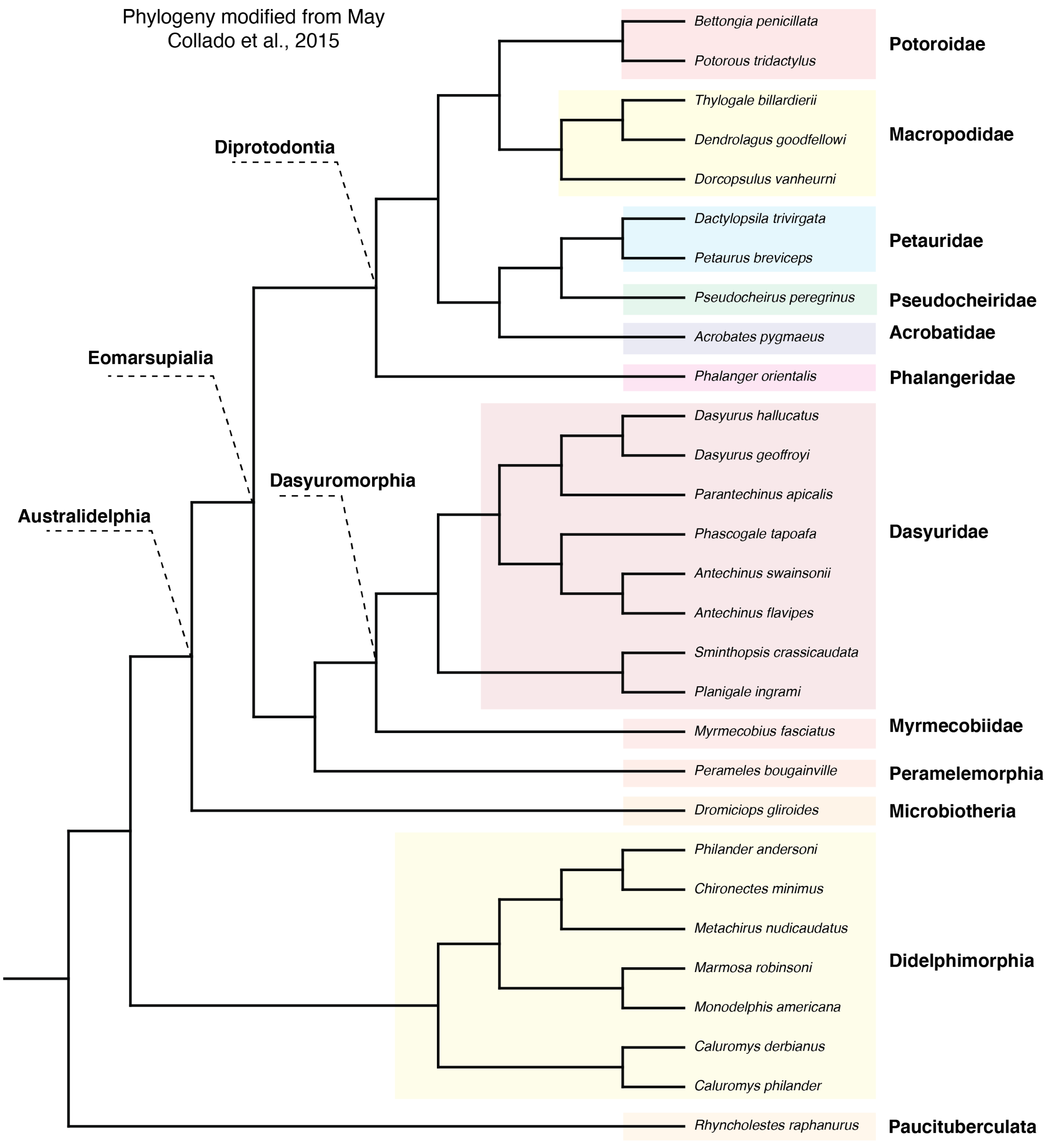

Figure 3: Species level phylogeny of the marsupial taxa used in this study. Phylogeny modified from May Collado et al.[16]

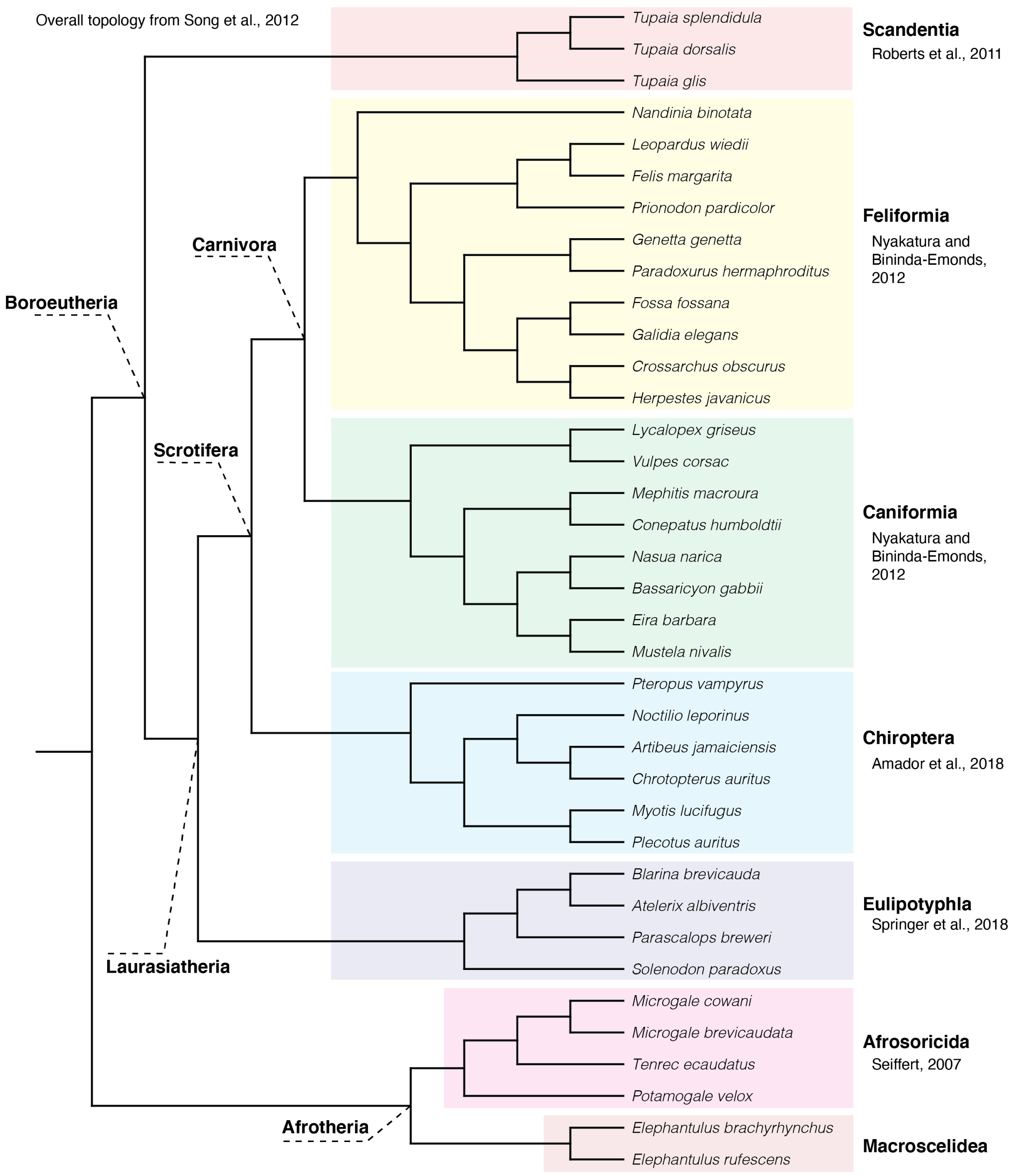

Figure 4: Species level phylogeny of the placental taxa used in this study. Phylogeny assembled from Song et al.[17] (overall topology), Roberts et al.[18] (Scandentia), Nyakatura and Bininda-Emonds[19] (Carnivora), Amador et al.[20] (Chiroptera), Springer et al.[21] (Eulipotyphla), Seiffert[22] (Afrosoricida).

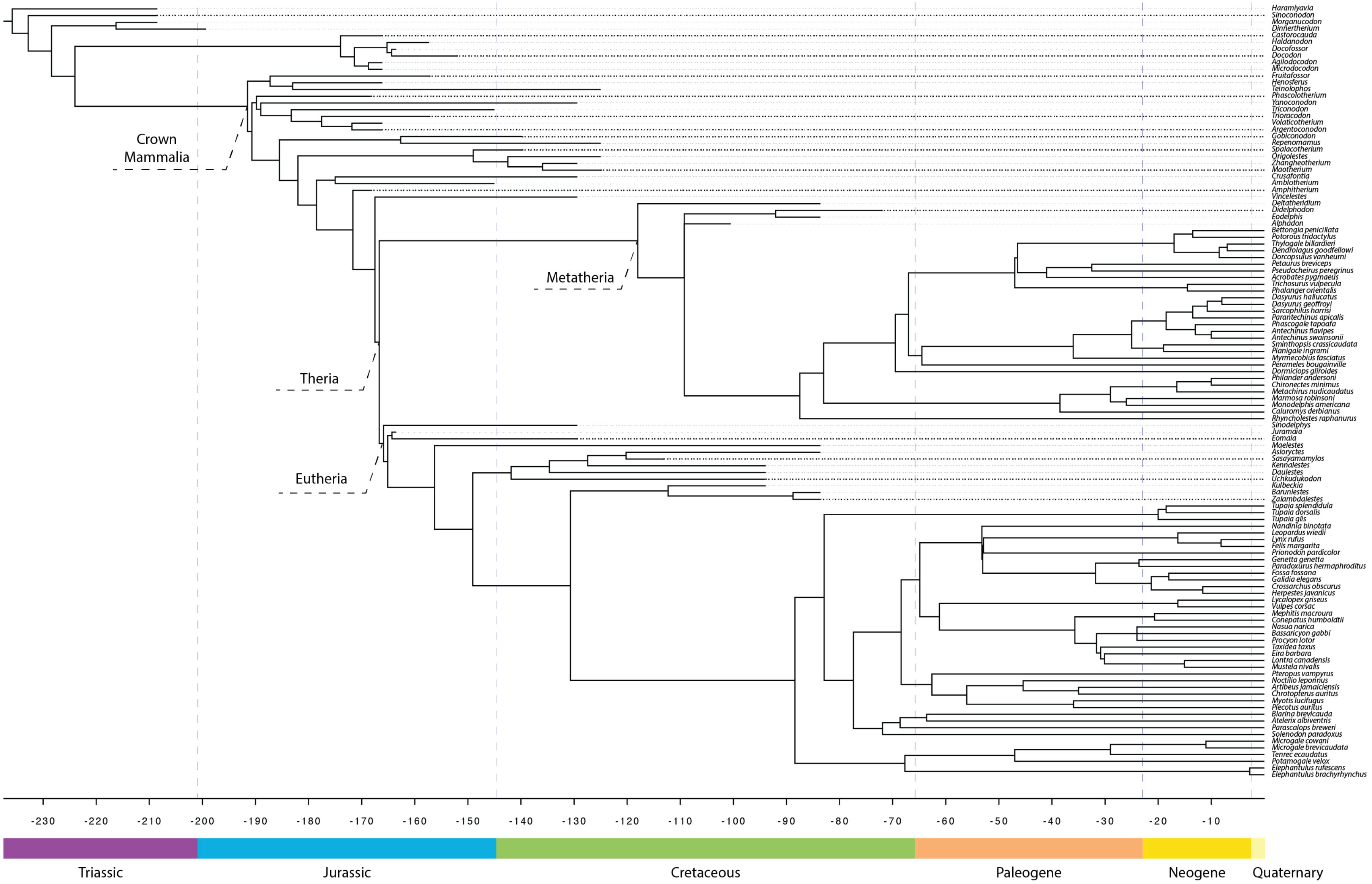

Figure 5: Time scaled phylogeny. Dated using the 'equal' method of Brusatte et al.[23]. Phylogeny assembled using sources detailed in Supplementary Figs. 2, 3, 4

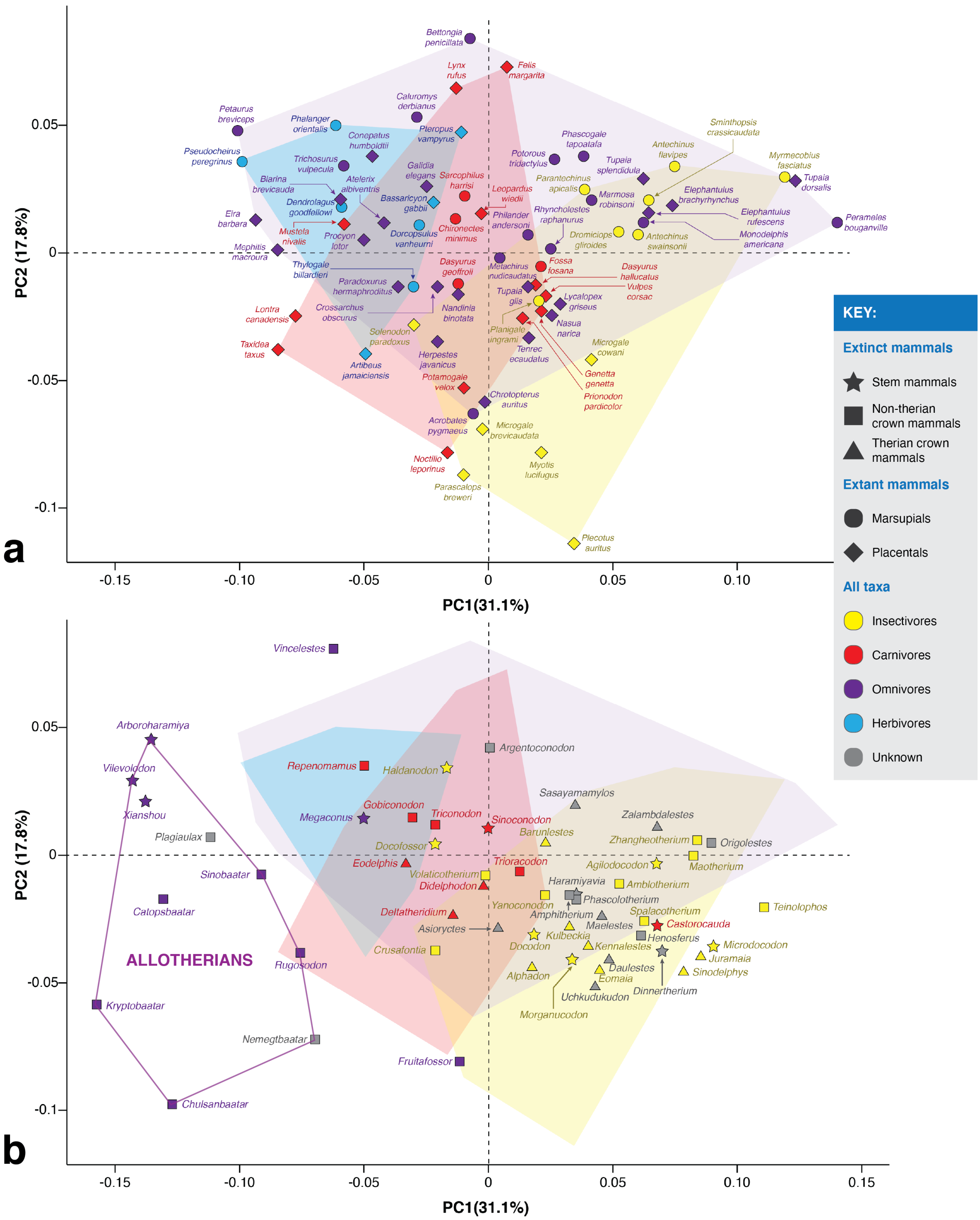

Figure 6: Scatter plots of the Principal Component Analysis (PCA) results (PC1 vs PC2), including allotherians (i.e., multituberculates and haramiyids). a) Extant taxa, b) Extinct taxa. Convex hulls shown for extant insectivores (yellow), carnivores (red), omnivores (purple) and herbivores (blue). Icon colours indicate known dietary categories of extant mammals and suggested dietary categories for Mesozoic mammals (obtained from the literature).

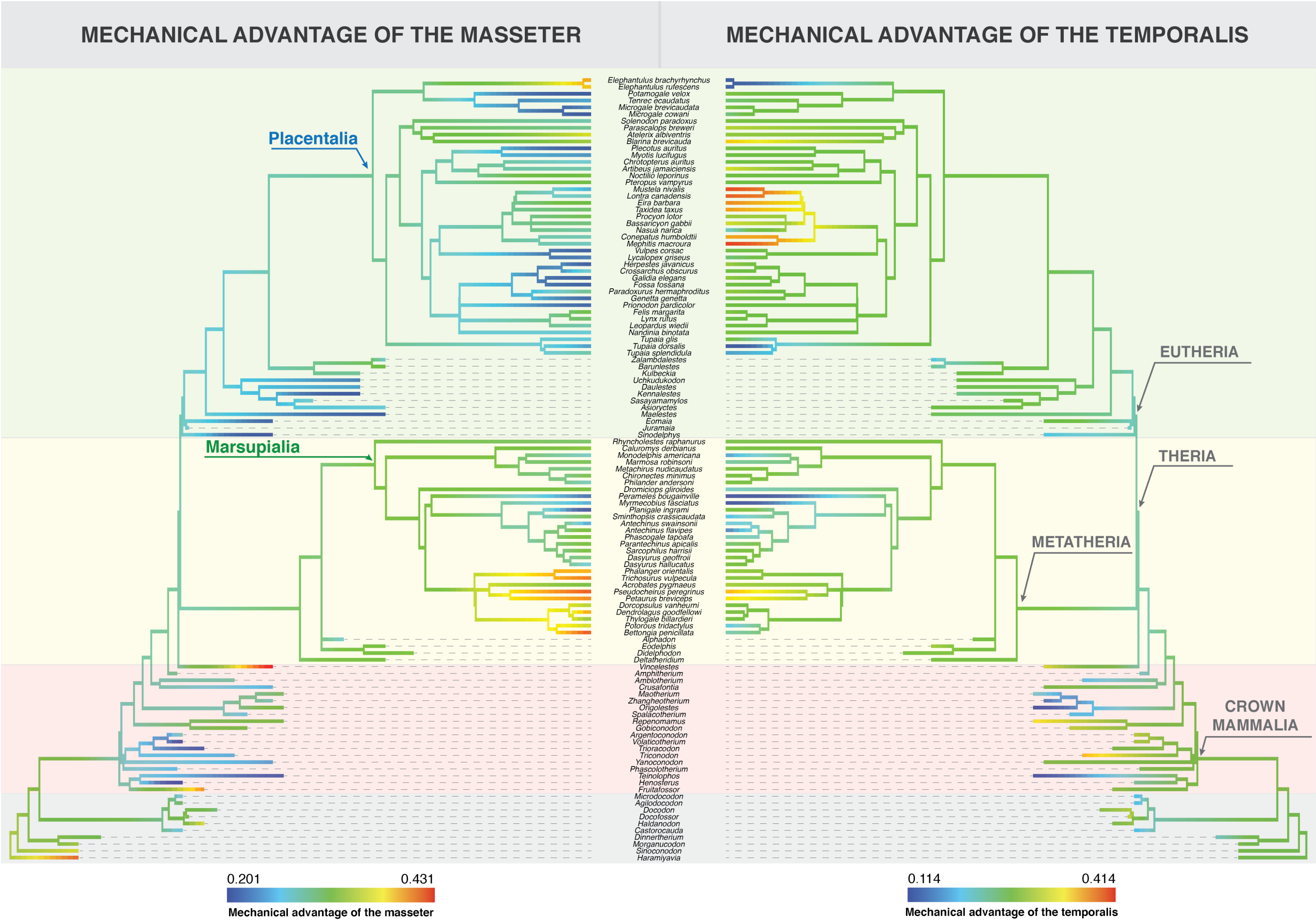

Figure 7: Mechanical advantage values of the temporalis (left) and masseter (right) visualised in the context of the phylogeny used in this study, with taxa names. Moment arm of resistance measured at the jaw tip.

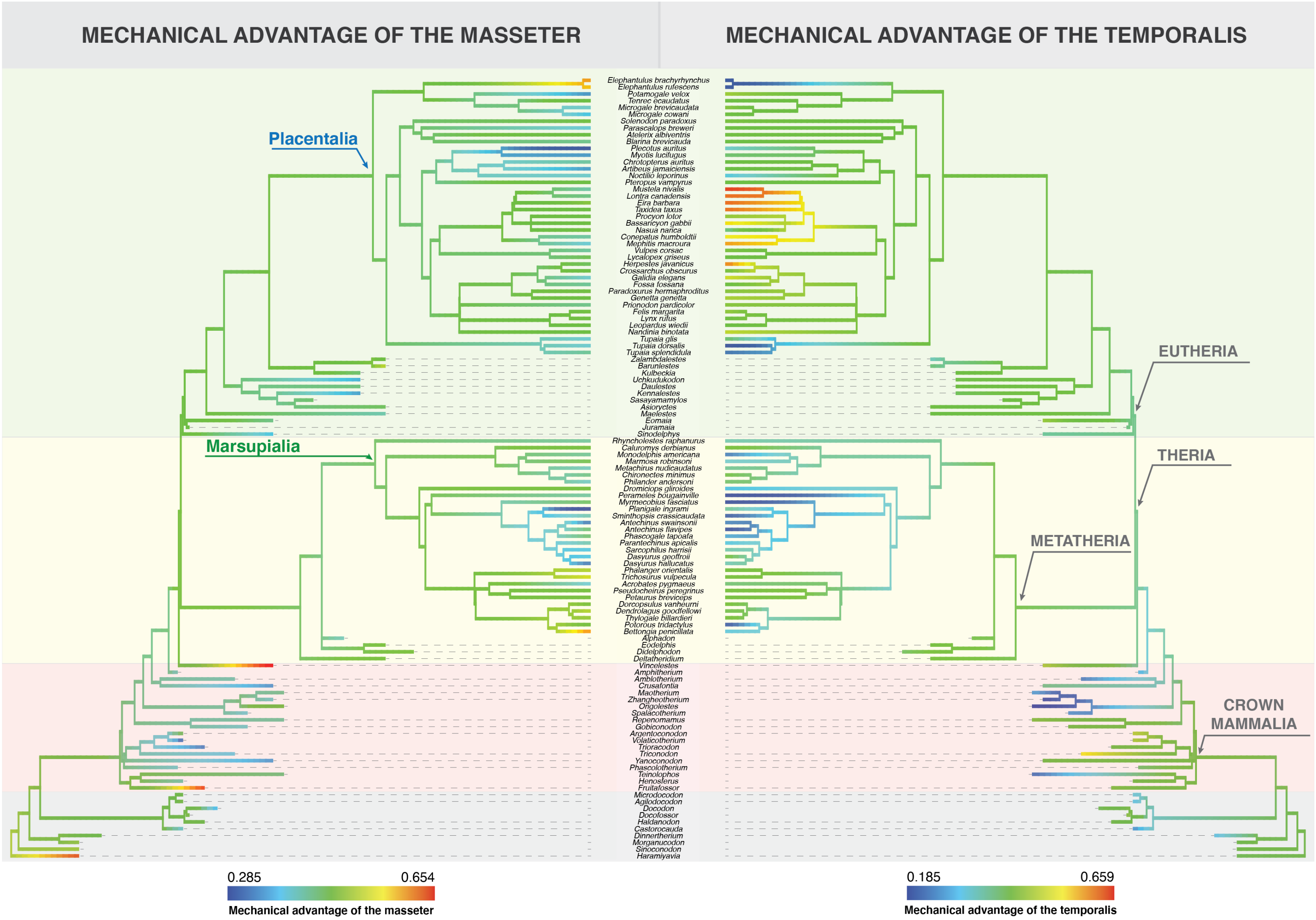

Figure 8: Mechanical advantage values of the temporalis (left) and masseter (right) visualised in the context of the phylogeny used in this study, with taxa names. Moment arm of resistance measured at the m1.

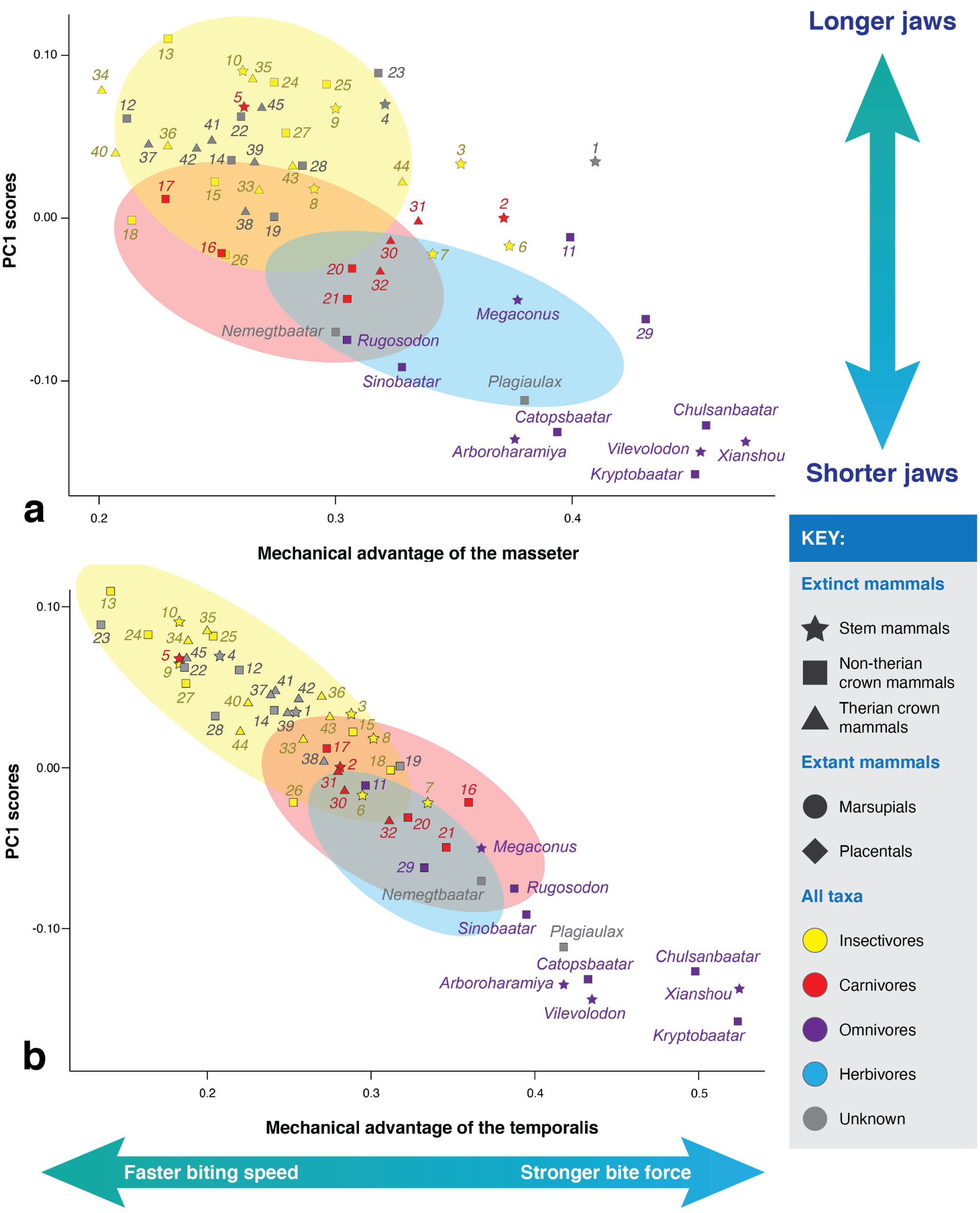

Figure 9: Scatter plot of the mechanical advantage of the adductor muscles (x axis) vs PC1 scores from Fig. 3 of the main text (y axis), which mainly describes jaw length. a) Mechanical advantage of the masseter, b) Mechanical advantage of the temporalis. Outlever measured at the jaw tip. Includes allotherians (indicated by genus names). Numbers of non-allotherians as in Table 1 of the main text. Colours indicate known dietary categories of extant mammals and suggested dietary categories for Mesozoic mammals (obtained from the literature). Ovals indicate where extant taxa of known dietary categories plot, as in figures 6 and 7 of the main text.

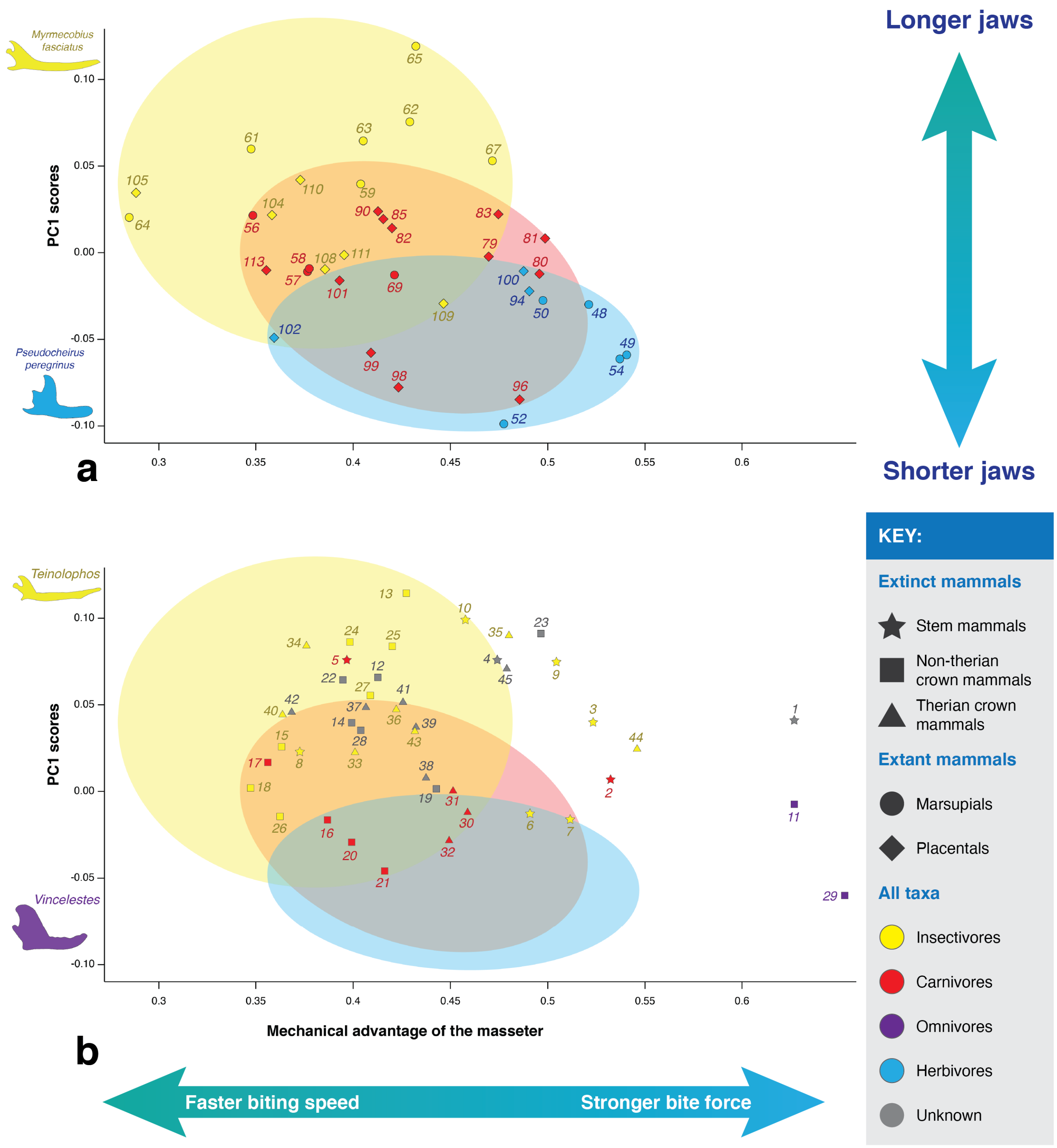

Figure 10: Scatter plot of the mechanical advantage of the masseter (x axis) vs PC1 scores from Fig. 3 of the main text (y axis), which mainly describes jaw length. a) Extant taxa, b) Extinct taxa. Outlever measured at the first lower molar (m1). Colours indicate known dietary categories of extant mammals and suggested dietary categories for Mesozoic mammals (obtained from the literature). Numbers as in Table 1 of the main text. Ovals indicate where extant taxa of known dietary categories plot, as in part (a).

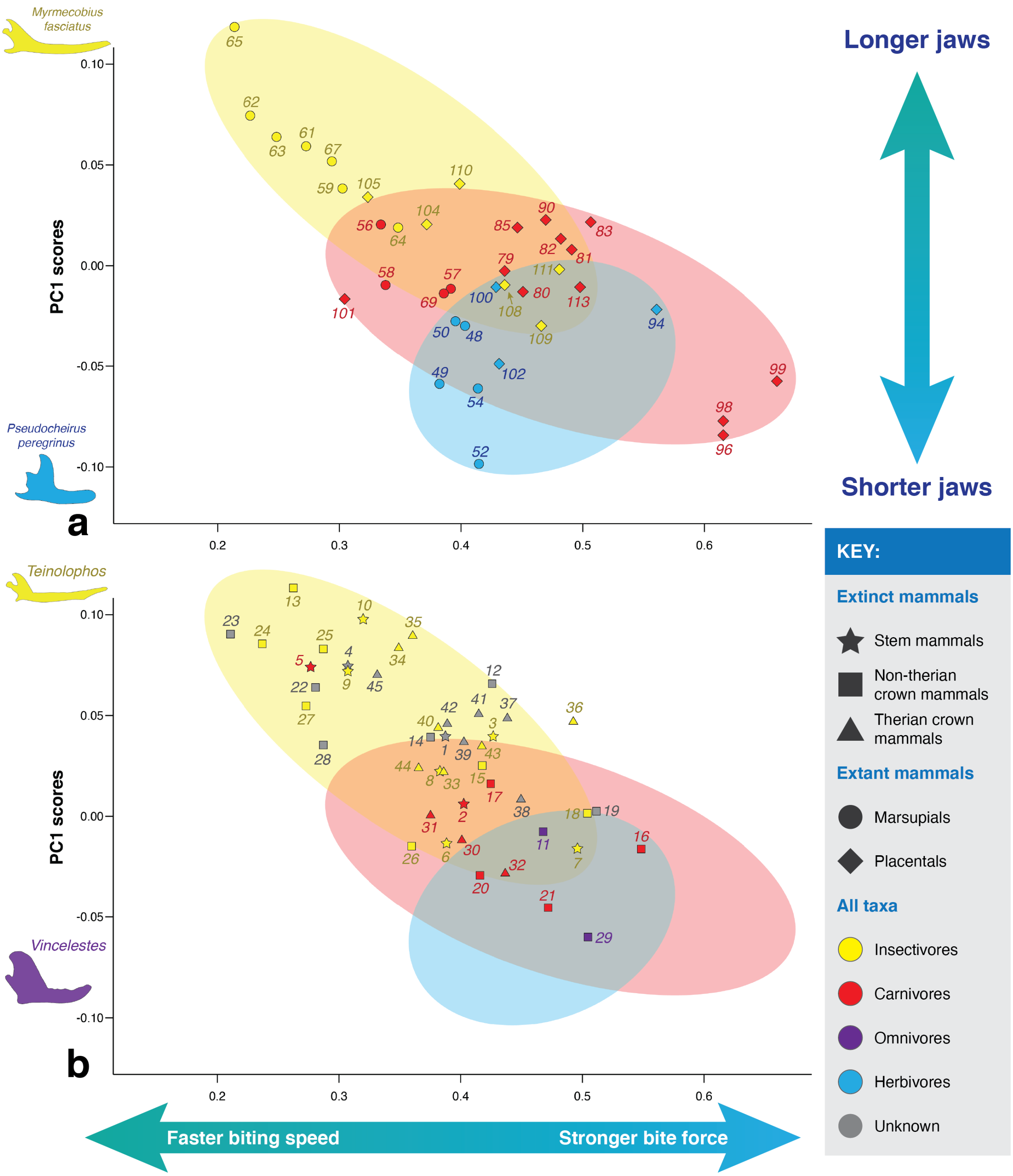

Figure 11: Scatter plot of the mechanical advantage of the temporalis (x axis) vs PC1 scores from Fig. 3 of the main text (y axis), which mainly describes jaw length. a) Extant taxa, b) Extinct taxa. Outlever measured at the first lower molar (m1). Colours indicate known dietary categories of extant mammals and suggested dietary categories for Mesozoic mammals (obtained from the literature). Numbers as in Table 1 of the main text. Ovals indicate where extant taxa of known dietary categories plot, as in part (a).

Morphofunctional landscapes

Jaw shape and mechanical advantage (outlever measured at the jaw tip and m1) were plotted together in morphofunctional landscapes (Figs. 12 and 13) in MATLAB R2019a 9.6.0 (The MathWorks, Inc., Natick, Massachusetts) following a protocol from Dr. J. A. Bright and previously used in Navalón et al.[24].

Our results show that generally, shorter jaws (negative PC1) have higher mechanical advantage and longer jaws have lower mechanical advantage, as predicted by the lever-like nature of the system, when measuring the outlever either at the jaw tip (Fig. 12) or the first lower molar (Fig. 13). There is a larger range of MA values when measuring the outlever at the m1 (MAM:0.285-0.645, MAT:0.185-0.659) than when measuring it at the jaw tip (MAM:0.201-0.431, MAT:0.114-0.414), but both plots largely follow the same pattern. We can see that taxa with negative PC1 scores (i.e., those with shorter jaws and high MA) are largely herbivores and carnivores, while taxa with positive PC1 scores (i.e, those with longer jaws and low MA) are typically insectivores (Figs. 12a and 13a). In the case of the masseter, taxa with negative PC1 scores (i.e., short jaws) and positive PC2 scores (i.e., tall ascending rami), tend to have the highest MAM values. In the case of the temporalis, MAT increases towards the negative end of PC1 (i.e., shorter jaws).

Mesozoic mammals (depicted with white icons) largely plot in areas of morphospace with low to intermediate MAM and MAT, with some exceptions (Figs. 12b and 13). For example, *Vincelestes* has some of the highest MAM and MAT values, congruent with an omnivorous diet consisting of meat and hard plant matter (or possibly durophagy)[25,26], and putative carnivorous taxa, such as *Triconodon* and *Repenomamus*, have high MAT values congruent with their proposed diets[6,27]. Also note that stem mammals (white star icons), most of which are considered insectivores, have higher MAM values than many other insectivores.

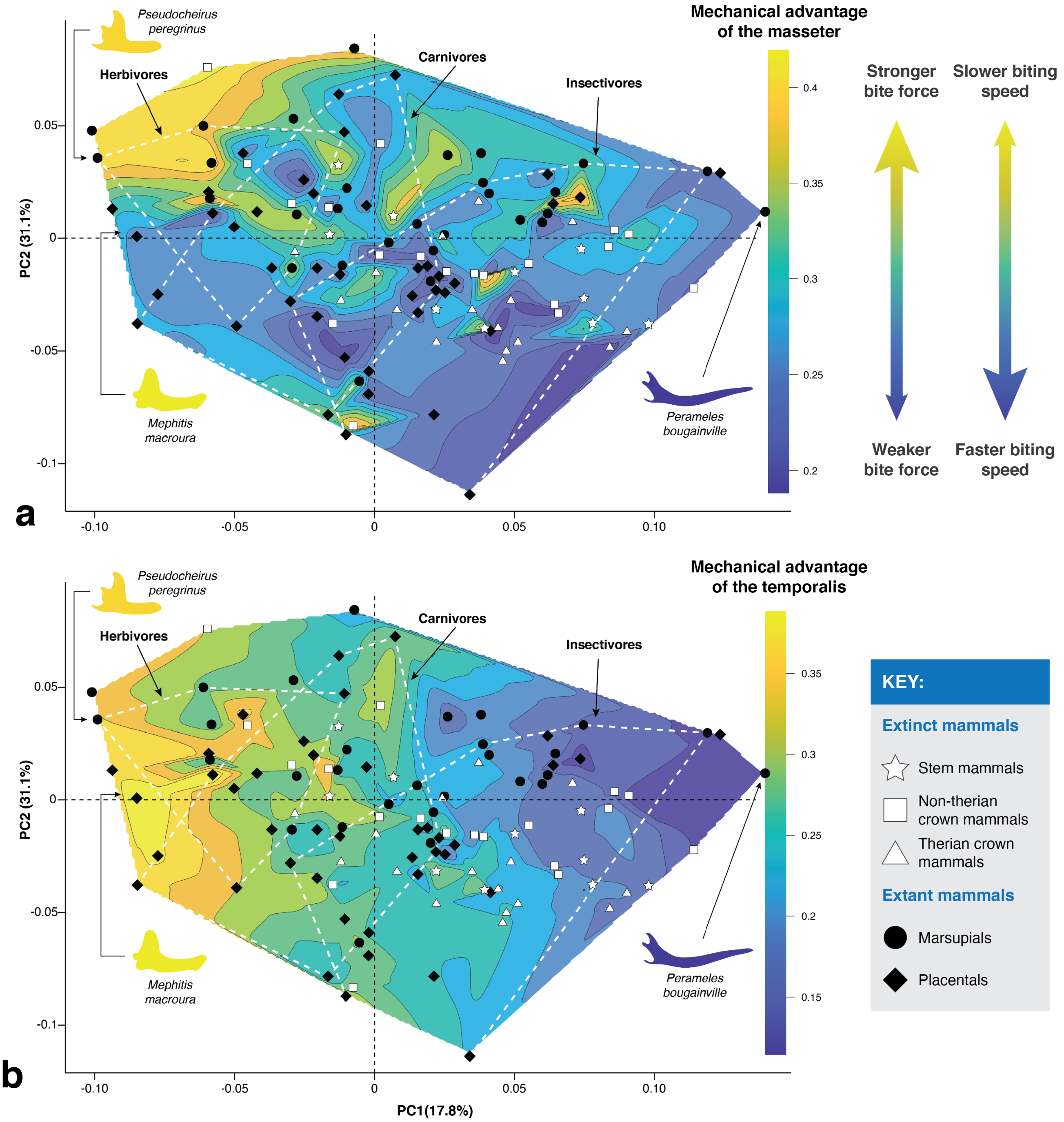

Figure 12: Morphofunctional landscape comparing a functional metric (i.e., mechanical advantage of the masseter [a, MAM] and temporalis [b, MAT] when biting at the anterior end of the jaw) with jaw shape (PC1 and PC2 axes). Outlever measured at the jaw tip. Silhouette colours are indicative of the mechanical advantage values of the taxa. Convex hulls in dashed lines as in Fig. 3 of the main text

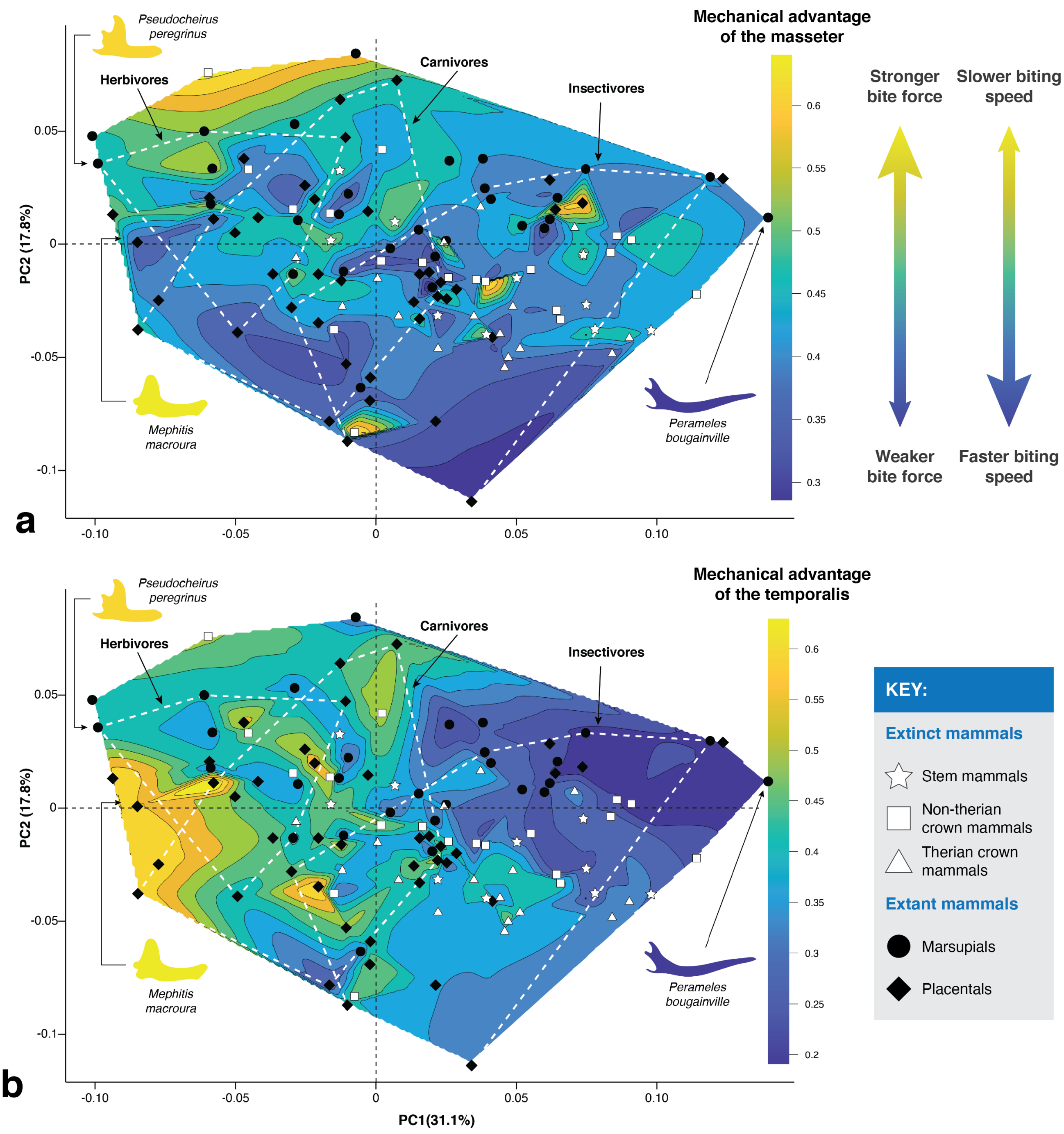

Figure 13: Morphofunctional landscape comparing a functional metric (i.e., mechanical advantage of the masseter [A, MAM] and temporalis [B, MAT]) with jaw shape (PC1 and PC2 axes). Moment arm of resistance for mechanical advantage measured at the m1. Silhouette colours are indicative of the mechanical advantage values of the taxa.

References

1. Close, R. A., Friedman, M., Lloyd, G. T. & Benson, R. B. Evidence for a mid-Jurassic adaptive radiation in mammals. *Current Biology* **25,** 2137–2142 (2015).
2. Huttenlocker, A. K., Grossnickle, D. M., Kirkland, J. I., Schultz, J. A. & Luo, Z. X. Late-surviving stem mammal links the lowermost Cretaceous of North America and Gondwana. *Nature* **558,** 108–123 (2018).
3. King, B. & Beck, R. M. Tip dating supports novel resolutions of controversial relationships among early mammals. *Proc. Roy. Soc. B* **287,** 20200943 (2020).
4. Meng, Q. J. *et al.* An arboreal docodont from the Jurassic and mammaliaform ecological diversification. *Science* **347,** 764–768 (2015).
5. Zhou, C. F., Bhullar, B. A. S., Neander, A. I., Martin, T. & Luo, Z. X. New Jurassic mammaliaform sheds light on early evolution of mammal-like hyoid bones. *Science* **365,** 276–279 (2019).
6. Rougier, G. W., Martinelli, A. G., Forasiepi, A. M. & Novacek, M. J. New Jurassic Mammals from Patagonia, Argentina: A Reappraisal of Australosphenidan Morphology and Interrelationships. *Am. Mus. Nov.* **3566,** 1 (2007).
7. Luo, Z. X. Transformation and diversification in early mammal evolution. *Nature* **450,** 1011–1019 (2007).
8. Gaetano, L. C. & Rougier, G. W. New materials of *Argentoconodon fariasorum* (Mammaliaformes, Triconodontidae) from the Jurassic of Argentina and its bearing on triconodont phylogeny. *J. Vertebr. Paleontol.* **31,** 829–843 (2011).
9. Mao, F. *et al.* Integrated hearing and chewing modules decoupled in a Cretaceous stem therian mammal. *Science* **367,** 305–308 (2020).
10. Averianov, A. O., Martin, T. & Lopatin, A. The oldest dryolestid mammal from the Middle Jurassic of Siberia. *J. Vertebr. Paleontol.* **34,** 924–931 (2014).
11. Williamson, T. E., Brusatte, S. L., Carr, T. D., Weil, A. & Standhardt, B. R. The phylogeny and evolution of Cretaceous-Palaeogene metatherians: Cladistic analysis and description of new early Palaeocene specimens from the Nacimiento Formation, New Mexico. *Journal of Systematic Palaeontology* **10,** 625–651 (2012).
12. Bi, S. *et al.* An Early Cretaceous eutherian and the placental-marsupial dichotomy. *Nature* **558,** 390–395 (2018).
13. Kusuhashi, N. *et al.* A new Early Cretaceous eutherian mammal from the Sasayama Group, Hyogo, Japan. *Proc. Roy. Soc. B* **280,** 20130142 (2013).
14. Archibald, J. D. & Averianov, A. O. Late Cretaceous asioryctitherian eutherian mammals from Uzbekistan and phylogenetic analysis of Asioryctitheria. *Acta Palaeontol. Pol.* **51,** 351–376 (2006).
15. Archibald, J. D., Averianov, A. O. & Ekdale, E. G. Late Cretaceous relatives of rabbits, rodents, and other extant eutherian mammals. *Nature* **414,** 62–65 (2001).
16. May-Collado, L. J., Kilpatrick, C. W. & Agnarsson, I. Mammals from 'down under': A multi-gene species-level phylogeny of marsupial mammals (Mammalia, Metatheria). *PeerJ* **3,** e805 (2015).
17. Song, S., Liu, L., Edwards, S. V. & Wu, S. Resolving conflict in eutherian mammal phylogeny using phylogenomics and the multispecies coalescent model. *Proceedings of the National Academy of Sciences of the USA* **109,** 14942–14947 (2012).
18. Roberts, T. E., Lanier, H. C., Sargis, E. J. & Olson, L. E. Molecular phylogeny of treeshrews (Mammalia: Scandentia) and the timescale of diversification in Southeast Asia. *Mol. Phylogenet. Evol.* **60,** 358–372 (2011).
19. Nyakatura, K. & Bininda-Emonds, O. R. Updating the evolutionary history of Carnivora (Mammalia): a new species-level supertree complete with divergence time estimates. *BMC Biology* **10,** 12 (2012).
20. Amador, L. I., Moyers Arévalo, R. L., Almeida, F. C., Catalano, S. A. & Giannini, N. P. Bat Systematics in the Light of Unconstrained Analyses of a Comprehensive Molecular Supermatrix. *J. Mammal. Evol.* **25,** 37–70. ISSN: 10647554 (2018).
21. Springer, M. S., Murphy, W. J. & Roca, A. L. Appropriate fossil calibrations and tree constraints uphold the Mesozoic divergence of solenodons from other extant mammals. *Mol. Phylogenet. Evol.* **121,** 158–165 (2018).

22. Seiffert, E. R. A new estimate of afrotherian phylogeny based on simultaneous analysis of genomic, morphological, and fossil evidence. *BMC Evolutionary Biology* **7,** 224 (2007).

23. Brusatte, S. L., Benton, M. J., Ruta, M. & Lloyd, G. T. Superiority, competition, and opportunism in the evolutionary radiation of dinosaurs. *Science* **321,** 1485–1488 (2008).

24. Navalón, G., Bright, J. A., Marugán-Lobón, J. & Rayfield, E. J. The evolutionary relationship among beak shape, mechanical advantage, and feeding ecology in modern birds. *Evolution* **73,** 422–435 (2019).

25. Bonaparte, J. *Sobre Mesungulatum houusayi y nuevos mamíferos Cretácicos de Patagonia, Argentina [On Mesungulatum houssayi and new Cretaceous mammals from Patagonia, Argentina].* in *Actas del IV Congreso Argentino de Paleontología y Biostratigrafía* (1986), 48–61.

26. Rougier, G. W. *Vincelestes neuquenianus Bonaparte (Mammalia, Theria) un primitivo mamífero del Cretacico inferior de la cuenca neuquina* PhD Thesis (Universidad de Buenos Aires, 1993).

27. Hu, Y., Meng, J., Wang, Y. & Li, C. Large Mesozoic mammals fed on young dinosaurs. *Nature* **433,** 149–152 (2005).
