# Supplementary material for: Jaw shape and mechanical advantage are indicative of diet in Mesozoic mammals
Source: Commun Biol. 2021 Feb 23;4:242. doi: 10.1038/s42003-021-01757-3 (PMC7902851; doi:10.1038/s42003-021-01757-3)
Supplement: Supplementary file 3 — Description of Additional Supplementary Files [file 42003_2021_1757_MOESM3_ESM.pdf]

Description of Additional Supplementary Files

File Name: Supplementary Data 1

Description: Spreadsheet that includes list of taxa, PC scores, mechanical advantage values (measured at jaw tip and m1), observed diet (extant mammals), proposed diet (extinct taxa), phylo FDA results (i.e., discriminant axis scores, predicted dietary class, and probability of belonging to a dietary group), first and last appearance dates, and references (photographs, diet and first and last appearance dates).
